# Optimizing substitution matrix choice and gap parameters for sequence alignment

**DOI:** 10.1186/1471-2105-10-396

**Published:** 2009-12-02

**Authors:** Robert C Edgar

**Affiliations:** 1 45 Monterey Dr, Tiburon, CA 94920, USA

## Abstract

**Background:**

While substitution matrices can readily be computed from reference alignments, it is challenging to compute optimal or approximately optimal gap penalties. It is also not well understood which substitution matrices are the most effective when alignment accuracy is the goal rather than homolog recognition. Here a new parameter optimization procedure, POP, is described and applied to the problems of optimizing gap penalties and selecting substitution matrices for pair-wise global protein alignments.

**Results:**

POP is compared to a recent method due to Kim and Kececioglu and found to achieve from 0.2% to 1.3% higher accuracies on pair-wise benchmarks extracted from BALIBASE. The VTML matrix series is shown to be the most accurate on several global pair-wise alignment benchmarks, with VTML200 giving best or close to the best performance in all tests. BLOSUM matrices are found to be slightly inferior, even with the marginal improvements in the bug-fixed RBLOSUM series. The PAM series is significantly worse, giving accuracies typically 2% less than VTML. Integer rounding is found to cause slight degradations in accuracy. No evidence is found that selecting a matrix based on sequence divergence improves accuracy, suggesting that the use of this heuristic in CLUSTALW may be ineffective. Using VTML200 is found to improve the accuracy of CLUSTALW by 8% on BALIBASE and 5% on PREFAB.

**Conclusion:**

The hypothesis that more accurate alignments of distantly related sequences may be achieved using low-identity matrices is shown to be false for commonly used matrix types. Source code and test data is freely available from the author's web site at http://www.drive5.com/pop.

## Background

Sequence alignment is a fundamental tool in contemporary biology. Most algorithmic formulations of the problem seek an alignment maximizing a function known as the objective score. Objective scores are usually defined as a sum of terms for matching pairs of letters (tabulated in a substitution matrix) and penalties for gaps. While the effects of different substitution matrices and gap parameters have been extensively studied in the context of local alignment and homolog recognition (see for example [1] and references therein), their effects on alignment accuracy, especially for global alignment, are not so well understood. Several heuristics are in common use, for example CLUSTALW's choice of low-identity matrices for aligning low-identity sequences [2], which have not to the best of my knowledge been empirically tested. One factor impeding such testing is the lack of effective automated methods for optimizing parameters for a given objective function. Previous work in this area has included unsupervised expectation maximization [3], discriminative training [4], inverse parametric alignment [5-9] and maximum-margin structured learning [10,11]. Katoh *et al*. [12] reported using golden section search to optimize gap penalties, but this generally assumes a unimodal function which does not hold in this case. Exhaustive search has been attempted several times, for examples see [1,13].

In this work I describe a new parameter optimization procedure, POP, compare it with the IPA method of [6], and use it to investigate a number of questions related to global protein alignment accuracy, including: which is the best substitution matrix, do the best choices of matrix and gap penalties vary with sequence identity, should terminal gaps be penalized differently from internal gaps, and does the loss of precision due to integer rounding in substitution matrices degrade alignment accuracy? IPA was chosen for comparison because it is a recently published method, code was readily available, and because it optimizes parameters exactly equivalent to those studied in this work. To the best of my knowledge there has been no prior comparative study of alternative gap optimization methods, so IPA's effectiveness relative to other approaches is not known. In the tests performed here, POP was shown to achieve from 0.2% to 1.3% higher training-set accuracy than IPA, and evidence is presented that POP finds parameters within about 0.1% of the true optimum, i.e. an improvement in training set error of up to an order of magnitude. Differences of a fraction of a percent will be shown to be informative in assessing differences between substitution matrices, for which a method with the improved precision of POP is required. POP, like IPA, is designed to maximize training set accuracy, which may be achieved at some cost in generalization error, i.e. worse performance on novel data. In the case of POP this is by design: the problem of minimizing training set error is simpler than minimizing generalization error and the experiments reported here were not designed to compute the most biologically appropriate parameters for a given algorithm but rather to compare models with small and equal numbers of free parameters. It is then reasonable to assume that training set accuracy is a good measure of the relative performance of those models.

**Figure 1:**
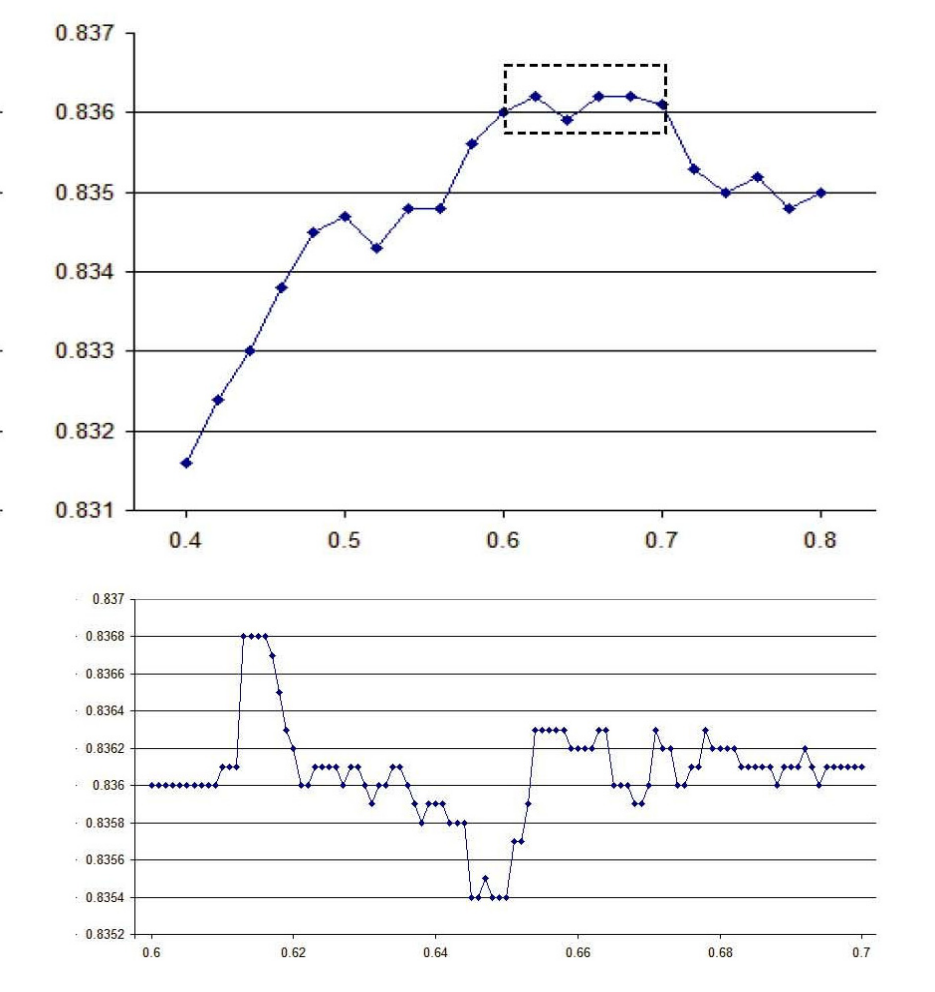
**A typical *Q *function**. The graphs show alignment accuracy (vertical axis) on all pairs in BALIBASE v2 as a function of extension penalty e (horizontal axis) with fixed gap-open penalty *g *= 6.5 and the BLOSUM70 matrix. The interval outlined in the dashed box on the left is expanded on the right. Note the many local maxima, and that trends are apparent with changes in *Q *of around 0.01 (left), while smaller changes are typically "noise". (right).

Let *w *= *w*_*i*_, *i *= 1...*N *be the parameters of interest (e.g., gap open and extend penalties), and *Q*(*w*) be the function to be optimized (e.g., an alignment accuracy or homolog recognition measure on some benchmark). The goal is to find values *w*_OPT _= *argmax*(*w*) *Q*(*w*) that maximize *Q*. This optimization problem is challenging for several reasons. Sufficiently small changes in *w *will leave all alignments unchanged and hence most *Q *functions of interest have zero partial derivatives almost everywhere. Also, *Q *is typically expensive to compute, requiring seconds to hours to evaluate at a single point, and is non-convex with many local maxima (Fig. 1).

## Methods

### IPA

Kim and Kececioglu [6] described an inverse parametric alignment method that can be trained on so-called "partial examples" such as those found in BALIBASE [14] in which only a subset of columns are annotated as reliably aligned. Eagu Kim (personal communication) kindly provided a software implementation of this algorithm (IPA). This implementation extended the method to allow separate terminal and internal gap penalties to be learned.

### POP

The central idea in POP is to search for changes in parameters that result in "Goldilocks" changes in *Q*--not too big, not too small, but changes in just of the right size to have a good chance of indicating coarse trends rather than small-scale noise. This approach can be regarded as a generalization of line search optimization (see for example [15]). As theoretical and empirical methods for dealing with non-convex, non-continuous *Q *functions are currently limited, practical experience, especially examination of plots such as Fig. 1, is the best guide to determining the size of a "just right" change. In the experiments reported here, values around 0.5% were most effective.

POP proceeds in three stages. In the first stage, an *N*-dimensional hypercuboid is explored by evaluating *Q*(*w*) at each point in a regularly spaced grid. Local maxima are identified as points at which *Q *is greater than all neighboring points, and the best of these are used as starting points for the second stage. (It is important to do this rather than to use the best *Q *values found in the grid because these will tend to cluster around the same maxima and it is better to find a diverse set of starting points to avoid local maxima.) The second and third stages use a hill-climbing strategy to approach a local maximum given a starting point. The second stage uses a faster but less accurate variant of the hill-climbing method than the third stage, which starts from the best local optima found in the second stage. The final result is the best maximum found by the third stage. To save computation time, the three stages typically use increasingly large subsets of the training samples, with the first two stages using randomly chosen subsets and the third stage the entire training set.

### Hill-climbing

Hill climbing repeats the following procedure until no improvement is found. Starting from a point *w*, each axis *i *is explored in both the positive and negative direction; i.e., all parameters are held fixed but one (*w*_*i*_). Let *δ *be a proposed change in *w*_*i*_,  = *w*_*i *_+ *δ*, *w' *= {*w*_*j*_, *j *≠ *i*, }, *Q' *= *Q*(*w*'), i.e. *Q' *is the value of *Q *after a proposed move along one axis, and *Δ *= *Q' *- *Q*. In each direction a *δ *is found that gives *Δ *> 0 (improves *Q*), or, failing that, a *δ *that reduces *Q *by an amount that is small, but not too small, say in the range 0.1% to 1%. Very small changes are undesirable because they will tend to be dominated by "noise" (Fig 1) rather than indicating a systematic trend. Large reductions in *Q *will tend to take *w*_*i *_out of the range where it might make an improvement when combined with a change along another axis (further discussed below). A heuristic designed to minimize these problems introduces a maximum (*μ*) and minimum (*λ*) reduction in *Q *(i.e., *λ *≤ -*Δ *≤ *μ*). After computing the proposed changes, a new w is determined as described in *Making a move *below.

### Proposed changes

A proposed change *δ *is determined as follows. In the first iteration *δ *is set to a small fraction (say, 10%) of the absolute value of *w*_*i *_or a small value (say, 0.1) if *w*_*i *_is zero, or a "hint" value set by the user. In subsequent iterations the initial value of *δ *is its final value from the previous iteration. Having set the initial value, the following procedure is then repeated. The value of *Δ *is computed. If *Δ *= 0, *δ *is multiplied by 4. If *Δ *< 0 and -*Δ *<*λ*, i.e. *Q *is reduced by too small an amount, *δ *is doubled. If *Δ *< 0 and -*Δ *>*μ*, i.e. *Q *is reduced by too much, *δ *is halved. Otherwise, *Δ *> 0 (*Q *is improved) and further exploration is tried. First, smaller values of *δ *(specifically, *mδ*/4 for *m *= 1, 2 and 3) are evaluated in an attempt to discover an intermediate maximum; if one is found this is repeated to investigate increasingly small changes. If smaller values give no further improvement, larger values are tried (*mδ*/4 for *m *= 5, 6, 7 and 8) in a similar way. A maximum improvement in *Q*, denoted *Γ*, is imposed in order to avoid extreme changes in a single parameter when far from an optimum (except when the smallest improvement found gives *Δ *>*Γ*, in which case it is accepted).

### Making a move

The search for proposed changes yields a set of 2*N *values {*δ*_*i*_+, *δ*_*i*_-, *i *= 1 ... *N*} where the + and - superscripts indicate moves in the positive and negative directions respectively. All moves are considered that apply the proposed changes on from *k *= 1, 2 ... *K *axes simultaneously, where *K *is a user-settable parameter may be 1, 2 ... *N*. When *k *> 1, new evaluations of *Q *are required. For example, *K *= 2 considers eight moves for each pair of parameters: (+, +), (+, 0), (+, -), (-, +), (-, 0), (-, -), (0, +), and (0, -) where + or -indicates an increase or decrease in the parameter and 0 indicates no change. The move giving the best improvement in *Q *is accepted, otherwise the routine terminates. Naively, one might expect that consideration of single-axis moves only (*K *= 1) would suffice, but in practice it turns out that allowing moves on two or more axes sometimes gives a significantly better final result, with *K *= 2 sufficient in most cases. It is desirable to keep *K *small as the number *d *of evaluated moves per iteration grows very rapidly with *K*:

It also turns out that allowing moves along axes that reduce *Q *can, when combined with moves on other axes, improve *Q *and that allowing this possibility also gives significantly improved optimization in some cases. For example, a small increase in the gap open penalty and a small decrease in the gap extend penalty might each reduce *Q*, but increase *Q *when both changes are made simultaneously.

### Fast hill-climbing

A "fast" variant of the hill-climbing procedure sets *K *= 1 (no multi-axis moves) and immediately applies any proposed move that is found to improve *Q*. Speed is also improved by increasing *μ*, *λ *and *Γ*. These modifications reduce the number of times the *Q *function is invoked, saving execution time but sometimes yielding significantly inferior parameters. Directions to try, and the sign (+ or -) first tried, may be selected at random or cycled to minimize systematic bias.

### Gap models and substitution matrices

Let a model be the set of parameters associated with a given objective function. In this work the substitution matrix is regarded as fixed; only gap penalty parameters are included. A gap is a series of indel symbols; formally, a maximal consecutive sequence of indels. If a gap includes the first or last column of an alignment it is described as terminal; all other gaps are internal. While POP has been implemented for a wide variety of models, most of these are works in progress and I will therefore report results for just two models: *g2*, in which the same affine penalties are applied to all gaps, and *g4*, in which internal and terminal gaps have different open and extend penalties. The parameters are *g*, *e*, *G *and *E*; the penalty for an internal gap of length *L *is *g *+ (*L *- 1) *e *and *G *+ (*L *- 1) *E *for a terminal gap. Thus *g2 *is a special case of *g4 *in which *G *= *g *and *E *= *e*. The objective score is then the sum of substitution scores minus gap penalties; a maximum-scoring global alignment is found using standard dynamic programming techniques.

The following substitution matrix types are considered: BLOSUM [16], RBLOSUM [17], PAM [18], JTT [19] and VT/VTML [20,21]. The RBLOSUM matrices were constructed using a bug-fixed version of the program used to compute BLOSUM matrices from the BLOCKS database [22]. Surprisingly, the corrected BLOSUM62 matrix was found to slightly degrade performance in homolog recognition [17]. Each matrix family is a series with members defined by a measure of evolutionary distance: percent identity cutoff in the case of BLOSUM and RBLOSUM, PAM distance for the rest. Conventionally, integer valued matrices are used in which log-odds scores in fractional bits have been rounded to one or two significant figures. Presumably this is for historical reasons: in older computer processors integer arithmetic was faster than floating point. This is no longer the case for many general-purpose processors, and regardless it is of interest to investigate whether the loss of precision due to integer rounding has an effect on alignment accuracy. Unless otherwise stated, full precision, one bit unit matrices were used.

### Benchmark data

Reference data was taken from three protein alignment benchmarks: PREFAB [23] version 4 and BALIBASE versions 2 and 3. There are 1,681 pair-wise reference alignments in PREFAB v4. Extracting all pairs from the multiple alignments in BALIBASE versions 2 and 3 gives 8,135 and 297,960 pairs respectively. The large number of pairs in version 3 motivated the use of the more tractable version 2 for all-pairs tests. Subsets 4 (alignments with long terminal gaps) and 5 (long internal gaps) of version 3 were used to investigate optimizing terminal gap penalties.

### Accuracy measure

The accuracy measure (*q*) for a single pair is the number of correctly aligned residue pairs divided by the number of residue pairs in the reference alignment. The total accuracy score (*Q*) is the weighted average of *q *over all pairs, where the weighting is uniform in the case of PREFAB and the inverse of the number of pairs in the original multiple alignment in the case of BALIBASE. It would have been desirable to estimate error bars using a method such as the Bayesian bootstrap [1]; however this proved to be infeasible due to limitations in available computer time (a CPU year was needed to generate the results reported here). For brevity, the reference sets will be denoted Bali (all-pairs from BALIBASE v2), Prefab (all-pairs from PREFAB v4), TermGaps (1,000 randomly selected pairs from BALIBASE v3, subset 4), and IntGaps (1,000 randomly selected pairs from BALIBASE v3, subset 5). To investigate the effects of evolutionary distance three subsets of BALIBASE v3 pairs were constructed: 1,000 randomly selected pairs with identities in the range 0-33% (Id0_33), 33-66% (Id33_66) and 66-99% (Id66_99), respectively. These were selected from the full-length rather than domain-trimmed sequences.

## Results

### Comparison of POP and IPA

I compared POP and IPA using the TermGaps set, where the biggest difference between g2 and g4 might be expected, and on Bali. The BLOSUM62 matrix in 1/3 bit units was used as this was hardcoded into IPA. Results are shown in Table 1. POP was found to be from 0.2% to 1.3% more accurate than IPA; these improvements are typical (additional results not shown).

**Table 1:** Training set accuracy

Set	Model	POP	IPA	>Acc
*Bali*	*g2*	80.3%	80.0%	0.4%

*Bali*	*g4*	80.6%	79.6%	1.3%

*TermGaps*	*g2*	80.2%	79.7%	0.6%

*TermGaps*	*g4*	84.0%	83.8%	0.2%

Training set accuracy (Q) for the *g2 *and *g4 *models on Bali and TermGaps using BLOSUM62 1/3-bit units. in The numbers in the POP and IPA columns are the Q values achieved by the gap para-meters reported by each method. In all cases, POP achieves higher accuracy, the >Acc column shows the amount by which the POP accuracy is better than IPA.

### Substitution matrix family

**Figure 2:**
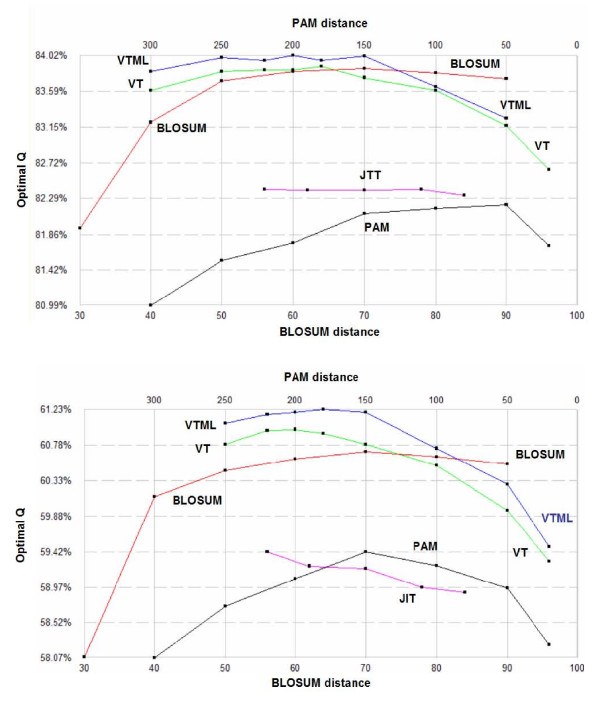
**Matrix accuracy**. Optimal accuracy (*Q*) according to POP for the VTML, VT, BLOSUM, JTT and PAM series using the g2 model on the Bali (upper) and Prefab (lower) training sets. The horizontal axis is the evolutionary distance of a matrix; the correspondence between PAM and BLOSUM distance is arbitrary.

**Figure 3:**
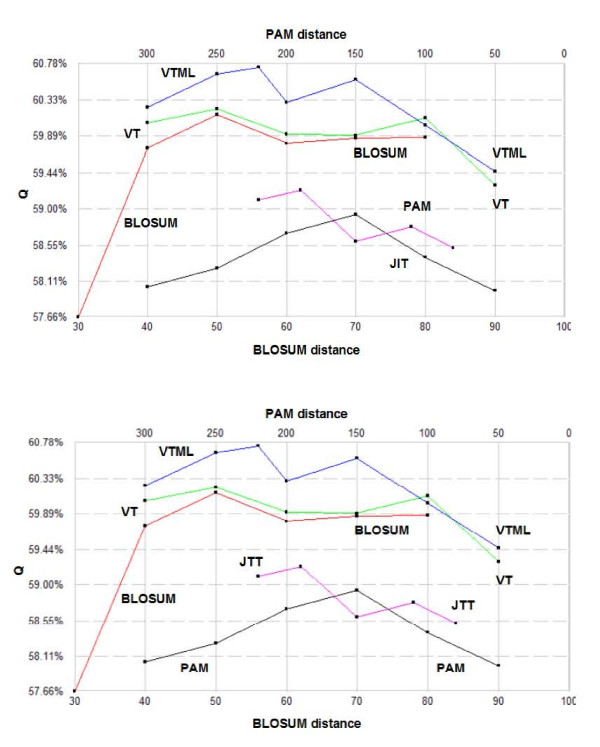
**Cross-training results**. Cross-trained results in which *g2 *parameters optimized for Bali were used to compute alignments on Prefab.

Fig. 2 shows the results of optimizing model g2 on the Bali and Prefab sets. The results are qualitatively similar on the two sets, giving confidence that they indicate general trends rather than artifacts of benchmark construction, of overtraining or of significantly suboptimal local maxima. This is further confirmed by cross-training (Fig. 3), which again gives similar, albeit noisier, results, as would be expected. The results show VTML to be the best matrix series, with VTML > VT > BLOSUM > JTT > PAM holding for most members, though the differences between VTML, VT and BLOSUM are small except at the extreme high and low-distance ends of the series. The PAM series is significantly inferior to VTML, giving accuracy scores around 2% lower.

### Matrix selection by evolutionary distance

It has been suggested that different substitution matrices might be more effective at different evolutionary distances. For example, CLUSTALW uses more distant matrices to align more distant sequences, and Lassmann and Sonhammer [24] report that "softer", i.e. more distant, matrices are better at aligning more more distant sequences while are equally good with more closely related sequences. To investigate this, I optimized *g2 *on the Id0_33, Id33_66 and Id66_99 sets, with the results shown in Fig, 4. Interestingly, the plots are qualitatively similar for the three sets despite increasing pair-wise identity and the increasingly narrow variation in optimal *Q*. Remarkably, in the case of Id66_99 accuracies are all above 99.5% and the difference between the best matrix (VT50) and worst (BLOSUM30) is only 0.17%, yet trends observed at lower identities are still clearly discernible. The peak in each curve that identifies the best matrix in each family is at approximately the same evolutionary distance on each set, showing that the best choice of matrix is almost independent of sequence divergence. Similar results are found with *g4 *(not shown), suggesting that the best matrix is also approximately independent of the gap model, as might be expected.

**Figure 4:**
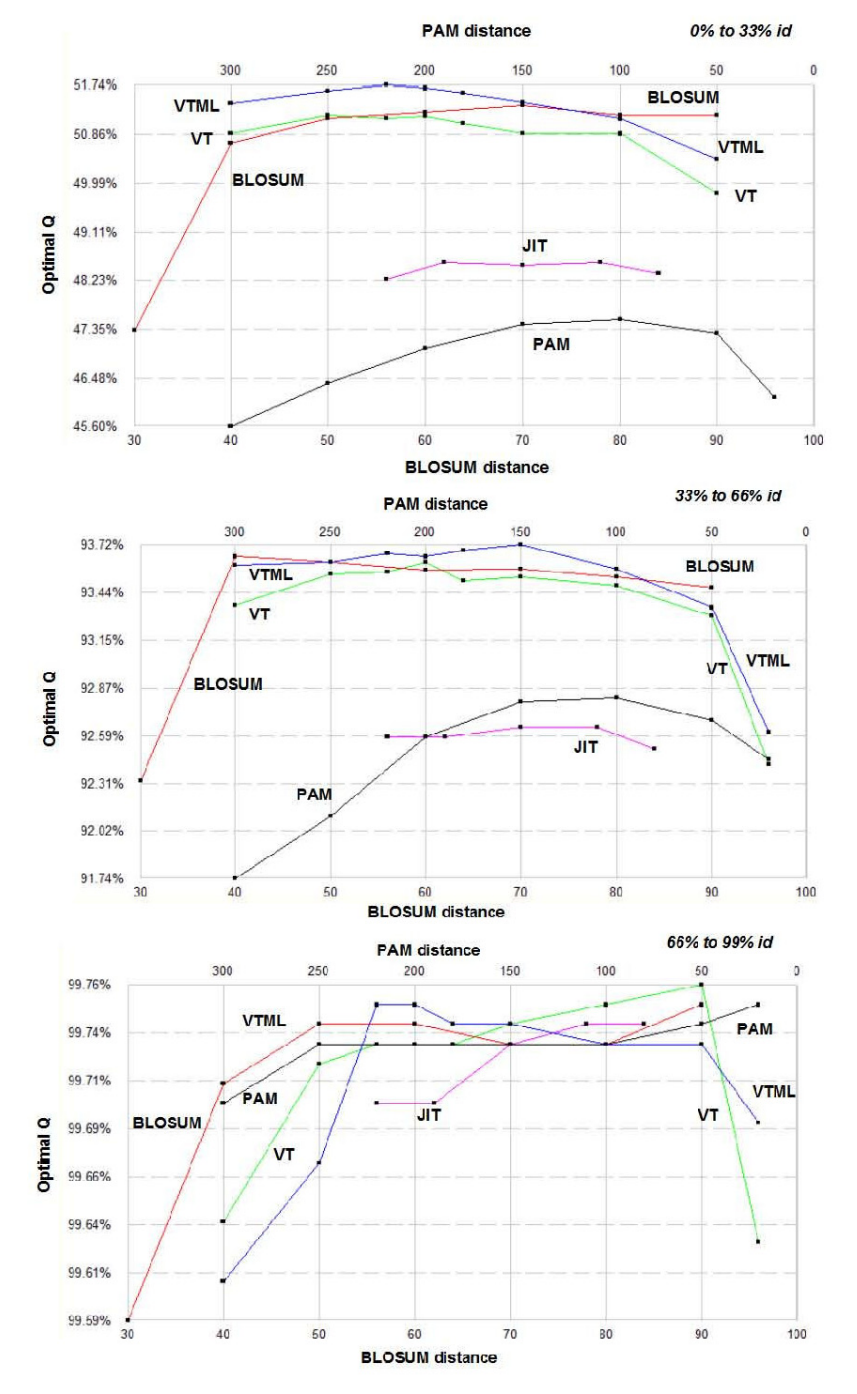
**Subsets by sequence identity**. Results for the *Id0_33 *(top), *Id33_66 *(middle) and *Id33_99 *(bottom) sets. Observe that the curves on the three sets are qualitatively similar with no evident trend for the peak in a given family to move towards higher or lower identities.

### Recommended matrix choice

In all the tests reported here, and others not shown, VTML200 is the best or close to best choice and is recommended as the general-purpose choice. In the VT and BLOSUM series, VT200 and BLOSUM70 respectively are recommended. In the clearly inferior PAM series, PAM100 to PAM150 appear to be best.

### Improving CLUSTALW accuracy

The above results lead to the conclusion that CLUSTALW's heuristic use of distance-dependent matrices is probably not effective and suggest that a superior strategy would be to use a single high-accuracy matrix. I tested this in CLUSTALW v2.0.9 [25] by setting the matrix to VTML200. This was necessarily an integer-rounded version as floating-point matrices are not supported by the software; I chose to use 1/3-bit units. The gap-open penalty was set to 2.0 and gap-extend to 0.1. These penalties were chosen after trying a few reasonable values; no attempt was made at systematic optimization. Results are shown in Table 2. An 8% improvement is seen in the column score on BALIBASE v3. This suggests that a small reduction in pair-wise alignment errors due to a better choice of substitution matrix can have a cumulative effect in a multiple alignment and yield a substantial improvement. However, it is not sufficient to raise the accuracy of CLUSTALW to those of more recent methods such as MUSCLE [23,26] or PROBCONS [3], as shown in Table 2.

**Table 2:** Improvement in CLUSTALW using VTML200

Test	Defaults	VTML200	>Acc	MUSCLE	PROBCONS
B3 TC	44.8%	48.4%	8%	53.2%	61.3%

B3 SPS	78.6%	80.9%	3%	84.4%	88.3%

P4 SPS	61.7%	64.6%	5%	67.7%	71.7%

Improvement in accuracy of CLUSTALW v2.0.9 by using the VTML200 matrix. B3 is BALIBASE v3, P4 is PREFAB v4. TC and SPS are the average fraction of correctly aligned columns and residue pairs, respectively. In PREFAB reference alignments are pair-wise so SPS and TC are equivalent. Defaults is the accuracies using default parameters, VTML200 with that matrix and gap parameters given in the main text, and >Acc is the improvement due to VTML200. Accuracies of the more recent methods MUSCLE v3.7 and PROBCONS v1.12 are given for comparison.

### Terminal gaps

The *g4 *model allows terminal gaps to be assigned different open and extend penalties versus internal gaps (see results in Table 3). On Bali and Prefab optimized terminal gap open penalties are roughly half those of internal penalties, validating the scheme used in MAFFT [12] and MUSCLE in which terminal gaps have the same extension penalty as internal gaps and half the open penalty. However, these results should be interpreted with caution since sequences in both Bali and Prefab are trimmed to domain boundaries. Multi-domain proteins are common, and different parameters and indeed different alignment algorithms may be appropriate when sequences have different domain organizations and are thus not globally alignable [27].

**Table 3:** Example parameters

Set	Model	*g*	*e*	*G*	*E*	*Q*
*Bali*	*g2*	5.370	0.423			80.7%

*Bali*	*g4*	5.874	0.473	2.332	1.488	81.0%

*Prefab*	*g2*	6.914	0.320			61.2%

*Prefab*	*g4*	6.742	0.476	3.964	0.322	62.3%

*TermGaps*	*g2*	6.508	0.133			80.8%

*TermGaps*	*g4*	5.010	0.635	15.507	0.026	84.2%

*IntGaps*	*g2*	5.725	0.304			78.3%

*IntGaps*	*g4*	5.725	0.304	6.419	3.343	78.4%

Optimized gap parameters reported by POP for the Bali, Prefab, TermGaps and IntGaps sets. Models are *g2 *(affine gaps) and *g4 *(different affine penalties for internal and terminal gaps). Penalties are *g *(gap-open), *e *(gap-extend), *G *(terminal gap-open) and *E *(terminal gap-extend). *Q *is the accuracy score. The matrix was the full-precision, one-bit unit version of VTML200, one of the best matrices according to the tests reported here.

### Random element

POP has a random element due to the selection of random subsets of the training data in its first two stages. It is therefore of interest to investigate how results vary for the same training data when different random number seeds are used. I chose to do this using the TermGaps set as practical experience shows it to have the most challenging parameter landscape of the sets considered here. I ran POP ten times for the g4 model on this set and found the following characteristics of the resulting *Q *values: mean 0.8412, standard deviation 5.3 × 10-4, maximum 0.8419, minimum 0.8401. These results suggest a low sensitivity to subset selection and are consistent with finding the global optimum to within approximately three significant figures.

### Integer rounding

Integer rounding causes a small, but consistent, degradation in accuracy of around 0.1% to 0.3% in VTML and 0.05% to 0.1% in BLOSUM (detailed results not shown).

### Corrected BLOSUM matrices

RBLOSUM gave a small but again consistent improvement in accuracy over BLOSUM of around 0.1%. This result is surprising considering that the opposite effect was found when testing homolog recognition [17].

## Discussion

POP is an *ad hoc *algorithm without a strong theoretical foundation and has more heuristic parameters than one would ideally like. It does not scale well to larger numbers of parameters. Implementing and using POP requires an understanding of the input data and some trial and error. However, consistency of trends across different benchmarks, even when differences are very small, and consistency when run with different random number seeds combine to suggest that POP may find a global optimum to within approximately three significant figures on the pair-wise global alignment tests considered here, and regardless provide strong evidence that POP provides a sensitive test of differences between models with the same number of parameters. It is also readily parallelized and, unlike some other approaches, can easily be applied to multiple alignment. The hill-climbing in POP can start from parameters proposed by some other method, such as IPA, providing a lower bound on the training set error. It should be noted that the reduced training set error achieved by POP may come at some cost in generalization error; this is a topic for further study.

Compute resources required by POP are relatively modest (Table 4), needing memory well within the range of current commodity computers and times (including alignments) ranging from minutes to hours on the tests considered here. The modern VTML matrices proved to give the best accuracy, in agreement with studies of homolog recognition [1]. The VT and BLOSUM series were not far behind, except for low-identity BLOSUMs which performed relatively poorly on all tests, even when aligning low-identity sequences. The RBLOSUM series (bug-fixed BLOSUM) was marginally better, but still inferior to VTML. Full-precision matrices were also marginally better than the integer-rounded versions in common use. It is natural to expect that improved pair-wise alignment accuracy will lead to improved multiple alignments, and this is a direction that deserves further exploration. The accuracy of CLUSTALW was significantly improved by using VTML200. It is therefore of interest to review the matrices employed by other multiple aligners. MUSCLE uses VTML240, which is close to VTML200 on most tests and therefore appears to have been a fortuitous choice. MAFFT v2 used PAM250, a clearly suboptimal choice; possibly this partly explains the lower accuracy of MAFFT v2 relative to the similar algorithm of MUSCLE v3. It is not clear to me which matrices are used in more recent versions of MAFFT, though the authors report testing members of the BLOSUM and JTT series [12]. ALIGN-M [28] uses BLOSUM35. Given the rapid drop in accuracy observed between BLOSUM40 and BLOSUM30 on all benchmark tests, it seems likely that a significant improvement would result from using a different matrix. KALIGN [24] uses GONNET250 [29] which I found to perform comparably with VTML200 (results not shown). MUMMALS [30] and PROBCONS use BLOSUM matrices, suggesting the possibility of a small improvement in those programs by using VTML instead.

**Table 4:** Typical compute resources required by POP.

Set	Model	Time (mins.)	Memory (Mb)
*Bali*	*g2*	135	72

*Bali*	*g4*	840	72

*Prefab*	*g2*	27	24

*Prefab*	*g4*	68	24

*TermGaps*	*g2*	55	26

*TermGaps*	*g4*	192	27

*IntGaps*	*g2*	13	12

*IntGaps*	*g4*	61	25

This following table shows the CPU time (minutes on a 2.2 GHz 64-bit Athlon processor) and memory (Mb) used to compute the results shown in Table 3.

An examination of Fig. 2 shows that BLOSUM60-70 achieves almost the same accuracy as VTML150-200 on BALIBASE but is roughly 1% worse on PREFAB. This suggests that BALIBASE may be biased towards BLOSUM62 due to the use of sequence methods in alignment construction (87% of BALIBASE sequences have unknown structure, so are necessarily aligned by sequence alone). The source code for an example implementation of POP is available from the author's web site at http://www.drive5.com/pop. This is designed for optimization of the *g2 *and *g4 *models described here, but can be modified relatively easily for other models by replacing the appropriate *Q *and alignment functions.

As I can attest from an abundance of personal experience, manual optimization of alignment parameters is a tedious process that rarely leaves the practitioner feeling confident in the results. Having an automated method at one's experimentation with new and modified algorithms by enabling a relatively trustworthy and painless evaluation of their relative effectiveness. I plan to use POP to explore ideas for improved pair-wise and multiple global alignment algorithms.

## Conclusion

On the basis of this analysis, the VTML200 matrix is recommended as the most appropriate in general when global alignment accuracy is desired. My results suggest a bias in BALIBASE towards the BLOSUM series of matrices of around 1% in accuracy. While the effect is small, a bias of this magnitude could be significant in validations of multiple alignment methods because differences between the better methods on BALIBASE are of comparable size. Bias towards substitution matrices or gap penalty functions is not unexpected as only 13% of BALIBASE sequences have solved structures, and its alignments were therefore constructed mostly by the use of sequence rather than structure methods. Future studies of alignment accuracy should use data derived independently of sequence in order to avoid such biases.
